# Google Glass for Documentation of Medical Findings: Evaluation in Forensic Medicine

**DOI:** 10.2196/jmir.3225

**Published:** 2014-02-12

**Authors:** Urs-Vito Albrecht, Ute von Jan, Joachim Kuebler, Christoph Zoeller, Martin Lacher, Oliver J Muensterer, Max Ettinger, Michael Klintschar, Lars Hagemeier

**Affiliations:** ^1^PL Reichertz Institute for Medical InformaticsHannover Medical SchoolHannoverGermany; ^2^Department of Pediatric SurgeryHannover Medical SchoolHannoverGermany; ^3^Division of Pediatric SurgeryNew York Medical CollegeValhalla, NYUnited States; ^4^Trauma DepartmentHannover Medical SchoolHannoverGermany; ^5^Institute for Legal MedicineHannover Medical SchoolHannoverGermany

**Keywords:** Google Glass, forensic medicine, autopsy, postmortem examination, documentation

## Abstract

**Background:**

Google Glass is a promising premarket device that includes an optical head-mounted display. Several proof of concept reports exist, but there is little scientific evidence regarding its use in a medical setting.

**Objective:**

The objective of this study was to empirically determine the feasibility of deploying Glass in a forensics setting.

**Methods:**

Glass was used in combination with a self-developed app that allowed for hands-free operation during autopsy and postmortem examinations of 4 decedents performed by 2 physicians. A digital single-lens reflex (DSLR) camera was used for image comparison. In addition, 6 forensic examiners (3 male, 3 female; age range 23-48 years, age mean 32.8 years, SD 9.6; mean work experience 6.2 years, SD 8.5) were asked to evaluate 159 images for image quality on a 5-point Likert scale, specifically color discrimination, brightness, sharpness, and their satisfaction with the acquired region of interest. Statistical evaluations were performed to determine how Glass compares with conventionally acquired digital images.

**Results:**

All images received good (median 4) and very good ratings (median 5) for all 4 categories. Autopsy images taken by Glass (n=32) received significantly lower ratings than those acquired by DSLR camera (n=17) (region of interest: *z*=–5.154, *P*<.001; sharpness: *z*=–7.898, *P*<.001; color: *z*=–4.407, *P*<.001, brightness: *z*=–3.187, *P*=.001). For 110 images of postmortem examinations (Glass: n=54, DSLR camera: n=56), ratings for region of interest (*z*=–8.390, *P*<.001) and brightness (*z*=–540, *P*=.007) were significantly lower. For interrater reliability, intraclass correlation (ICC) values were good for autopsy (ICC=.723, 95% CI .667-.771, *P*<.001) and postmortem examination (ICC=.758, 95% CI .727-.787, *P*<.001). Postmortem examinations performed using Glass took 42.6 seconds longer than those done with the DSLR camera (*z*=–2.100, *P*=.04 using Wilcoxon signed rank test). The battery charge of Glass quickly decreased; an average 5.5% (SD 1.85) of its battery capacity was spent per postmortem examination (0.81% per minute or 0.79% per picture).

**Conclusions:**

Glass was efficient for acquiring images for documentation in forensic medicine, but the image quality was inferior compared to a DSLR camera. Images taken with Glass received significantly lower ratings for all 4 categories in an autopsy setting and for region of interest and brightness in postmortem examination. The effort necessary for achieving the objectives was higher when using the device compared to the DSLR camera thus extending the postmortem examination duration. Its relative high power consumption and low battery capacity is also a disadvantage. At the current stage of development, Glass may be an adequate tool for education. For deployment in clinical care, issues such as hygiene, data protection, and privacy need to be addressed and are currently limiting chances for professional use.

##  Introduction

### Background

Emerging technologies originally developed for the customer sector often find their way into professional environments. A prime example is the use of smartphones and tablet computers in combination with medicine-related apps in hospitals [1-3]. Google Glass [4] is another new and promising device originally developed with private consumers in mind that will soon be available for the general public. In principle, Glass is an optical head-mounted display that in addition to its technical capabilities also allows easy communication with various Internet-based services (mainly provided by Google). With some exceptions, its voice and gesture control functionalities allow an almost hands-free mode of operations. Using a prism, the display information is presented on the retina of the user and provides various kinds of information and visual feedback. The device is capable of taking pictures and recording videos by using an integrated camera. By using wireless access to the Internet, this information can be shared with the public. Also, because Glass provides a built-in microphone and a bone conduction transducer for audio signals, voice over Internet protocol (VoIP) communication is easily possible (eg, by using Google Hangout). Various sensors integrated into the device allow using it for rich augmented reality-based applications.

Currently, Glass is in a premarket state and only participants of the Glass Explorer Program have had the chance to evaluate the device so far [5], but some reports of proof of concept projects, especially in the medical field, are already available [6,7]. Lucien Engelen, based at Singularity University (Silicon Valley, CA, USA) and in Europe at Radboud University Medical Center, was the first health care professional in Europe to commence research on the usability and impact of Google Glass in the field of health care [8]. His collection of reports published since July 2013 comprises descriptions of experiences gained while using the device in a number of settings, including in operating theaters, ambulances, general practices, and other settings. His reports have been published primarily on social media channels [9].

At the moment, no empirical evidence for using Glass in a medical setting is known to the authors. A literature search on PubMed using the keywords “Google Glass” only resulted in a limited number of articles [10,11]. Nevertheless, Glass seems to be perfect for hands-free documentation purposes. Therefore, we were interested in the feasibility of integrating the device in a medical setting. We were also interested in usability aspects, including its effectiveness (can users successfully achieve their objectives?), efficiency (how much effort does it take to achieve those objectives?), and satisfaction (was the subjective experience satisfactory?). We decided to use Glass in a medical field where it was possible to evaluate the device with respect to these questions in a real setting while avoiding any risk to patients. The field of forensic medicine was selected as ideal for our purpose because photo documentation is of high value in this field.

### Forensic Medicine

**Figure 1:**
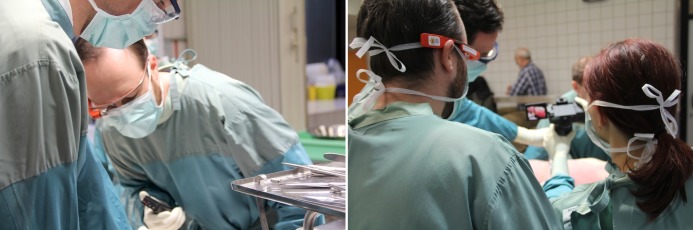
A typical crowded autopsy situation. On the left, the forensic pathologist is wearing Google Glass, allowing him to take pictures in a hands-free manner. On the right, the procedure is interrupted by an assistant taking a picture with a camera, which narrows the already limited space around the body.

There are a number of demands placed on documentation in forensics. The description must be unambiguous and of high diagnostic value, yet easy to comprehend for laypersons, especially in court proceedings [12]. Photographic documentation may make a significant contribution to this and is expressly called for in the American Forensic Autopsy Performance Standards [13]. Because a textual description of a specific situation, such as anatomical structures, wounds, or hematomas, often requires imagination as well as a good knowledge of anatomy, a photograph may provide valuable help and aid readers in interpretation of the findings, especially if a reference scale is used. During forensic examinations and in autopsies, photographs can be used to document different stages of preparation if the original situation cannot be preserved. Particular findings can be recorded easily, in a timely manner, and are also easily verifiable and cost-efficient because of digital photography. However, when using conventional digital cameras, somebody has to operate the camera and this person must have a minimum of knowledge in camera handling and in the basics of forensic pathology. Until a few years ago, professional photographers were employed for this purpose, but nowadays—at least in Germany—this work has been delegated to the medical examiners themselves for economic reasons. The main problem with this approach is that an examiner who is working on a corpse cannot take hands-free photographs (Figure 1). Therefore, one has to change gloves to take pictures, often multiple times during the procedure, which costs time and resources. In this context, a camera that could be operated hands-free while taking the pictures and capturing the details the examiner wants with an image quality comparable to a commonly used digital single-lens reflex (DSLR) camera would mark an innovation in this field.

### Postmortem Examination and Autopsy

Postmortem examinations can be traced far back throughout history. Although the most important reason to perform such examinations is to determine whether a person is really dead—otherwise resuscitation measures must be initiated—the cause and manner of death (eg, natural death, accident, suicide, murder) has always been of further interest [13,14].

During a forensic examination of deceased persons, the examiner looks for externally visible evidence that may provide a hint to the manner of death, such as injuries, and samples may also be taken. In Germany, there is also a legal requirement to perform a forensic examination on every corpse that is to be cremated. This is because most indications and traces as to the cause of death will be permanently lost after cremation. Still, the results of a forensic examination have their limitations and the cause of death determined in this manner will always just be a suspected diagnosis. A forensic examination will never be able to substitute for an autopsy or have its probative value.

Over 100 years ago, Rudolf Virchow urged doctors to follow a standardized procedure for autopsy cases, which is meant as a useful guidance [15]. Nowadays, this is commonly acknowledged, but an autopsy should not be pressed into a rigid scheme [13,16,17]. Still, it should consist of 2 components, specifically an outer and an inner autopsy [18]. After describing the general impression, a systematic description of all body parts is required [13,19]. For this purpose, the head, throat and neck, chest, abdominal wall, back, outer genitalia and anus, as well as the upper and lower extremities are surveyed. Only when this is completed is the inner autopsy started. In Germany, §89 StPO (Code of Criminal Procedure) stipulates that all body cavities (head, chest, and abdominal cavity) must be opened and inspected, even if the cause of death seems to already have been ascertained during earlier steps. Every individual organ has to be examined and dissected [13,19]. If necessary, medical implants or foreign bodies as well as fractures, hematomas, and the skin, may have to be dissected to answer specific additional questions. In other jurisdictions (eg, following the American Forensic Autopsy Performance Standards), autopsies are performed in a similar manner [13].

## Methods

### Image Acquisition

**Figure 2:**
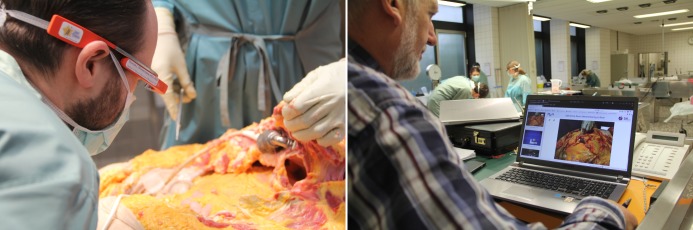
A forensic pathologist taking a picture of the situs using Google Glass during an autopsy (left). The Blink-app app transmits the image to the laptop where it is immediately presented to the attending detective and the public prosecutor (right).

We used Glass during one autopsy (Figures 1 and 2). The senior physician leading the autopsy was equipped with Glass and was instructed to perform the procedure following the previously described standards. Beforehand, the physician was trained in using the device and a self-developed app used for capturing images (which we called “Blink-app”), which took approximately 5 minutes in total. Because the device itself is very straightforward to use and the app only requires a single voice command to start and a nodding motion or a head shake (for keeping or deleting an image) once the image is taken, the physician stated he felt confident in using it after he had practiced taking images a few times.

In addition, 16 postmortem examinations of 4 cases were performed by 2 physicians with training in forensic medicine. Both physicians conducted their examinations alternately with Glass (8 examinations) and a DSLR camera (Olympus E-600, lens: Olympus *Z*uiko Digital ED 14-42 mm F3.5-5.6, 8 examinations) without using the internal flash of the camera. The kind of device and who began the examination on which case was randomized by running a random number generator (with numbers 0 and 1) 3 times: first, to determine who of the 2 examiners should start, the second time to determine the device to use for the first of the 2 examinations of that examiner. The third call was used to determine the order of devices for the second examiner. The forensic pathologists were asked to follow their standardized routine. Therefore, they were not required to take a specific number of pictures, but were allowed to take as many pictures as they deemed necessary for documentation purposes.

### Glass

The device—a Google Glass explorer version—available during our prestudy (performed at the beginning of December 2013) ran on Android 4.0.4 (XE11). At the time of the prestudy, the Glass development kit had become available to the general public, which enabled us to build applications using methods and tools already established for other Android-based devices. Specifications of the available developer explorer unit included a Texas Instruments open multimedia applications platform (OMAP) 4430 SoC 1.2G Hz Dual (ARMv7), a 5-megapixel camera, 682 MB of memory, and 16 GB of storage, of which 12 GB were available for user purposes, as well as Wi-Fi 802.11b/g and Bluetooth. Sensors, such as a gyroscope, an accelerometer, and a magnetometer (all for 3 axes), as well as an ambient light and proximity sensors were also integrated in the device [20].

### Software Setup

For image acquisition, we decided to refrain from using the preinstalled camera app for two reasons. First of all, without having performed an analysis of the data streams that occur whenever the device logs into a wireless network, we were unsure whether data acquired using the stock camera app would be relayed in any way to any third parties, such as Google. This would be understandable from their point of view given that the device available for our study was still at a premarket stage and they would want to obtain data about possible bugs. Nevertheless, although we were dealing with deceased persons, this would still have been problematic because of the sensitive nature, both ethically and legally, of the procedures that were performed. Secondly, at the time of our prestudy, running the preinstalled app completely hands free was problematic. Nevertheless, because of hygiene issues, this is a necessity in most medical environments, including forensic postmortem examinations and autopsies.

Therefore, using the available development kit, a native app was developed that alleviated these problems. This app, called Blink-app, made use of the standard camera application programming interface (API) provided by Google. It could be started through voice commands and used specific but easy to perform gestures, such as nodding or shaking the head (evaluated using rotation vectors acquired from the geomagnetic sensor), to allow the user to specify whether an image should be kept or deleted. Using functionality integrated into this app, all accepted images were stored in a separate folder on the device and were securely transmitted via Wi-Fi to a Linux-based laptop that served as a wireless hotspot for Glass, but did not have any connection to the Internet. On this laptop, an Apache server had been set up to accept and store images transmitted from the device through hypertext transfer protocol (HTTP) POST requests for later evaluation. Additionally, the server setup allowed other personnel to review the acquired images in a Web browser on the laptop’s (larger) screen immediately after they were taken. Image viewing could be performed in various scale modes ranging from an overview to zooming into the image (up to its original resolution) to allow onlookers to closely scrutinize specific areas. As soon as the procedures were over, any images remaining on the device were manually deleted.

### Usability and Acceptance

The 2 forensic pathologists involved in taking the images were interviewed about usability aspects of Glass in combination with the Blink-app app. We asked questions about general experience, usage, and handling of the device, noteworthy positive and negative aspects, and whether there were any surprises. Because only 2 examiners used Glass, we refrained from using standardized usability instruments.

### Subjective Ratings of Image Quality

A total of 6 forensic pathologists (3 male, 3 female; age range 23-48 years, mean age 32.8 years, SD 9.6), with mean work experience of 6.2 years (SD 8.5) were included in this evaluation after having given their informed consent for participation in the evaluation of the quality of the acquired images. The evaluation was done in two parts: the images were divided into 2 groups according to the settings they were taken in (autopsy and postmortem examination) and for each of the images, the device used for acquisition was noted (DSLR camera or Glass) in the internal database of the app we used for presenting and evaluating the images. All pictures were evaluated by all 6 participating forensic pathologists. To avoid differences in presentation, all used the same tablet computer with fixed display settings (maximum brightness and automatic adjustment for brightness had been deactivated) under similar lighting conditions. Also, during their evaluation, the raters were not informed about which of the two devices had been used for taking the presented images.

In step 1, 49 pictures of the autopsy were presented to the participants using a self-developed evaluation app. The images taken by both devices were intermixed and were then presented in a randomized manner on a single 10” Android-based tablet computer (Samsung Galaxy Note 10.1N).

The participants were presented each image sequentially and they had to give their opinion about whether they were satisfied with it with respect to 4 parameters:

1. Region of interest or; specifically, whether all necessary anatomical structures were depicted.
2. Sharpness.
3. Overall color setting; specifically, whether the images allowed adequate discrimination of even small changes in color which could be important to document hints about underlying pathologies (eg, bruises on a decedent’s skin). We were not interested in color cast caused by the devices.
4. Image brightness achievable based on the defined lighting installed in the autopsy room. Although one could argue that using a flash might have improved brightness of the images taken with the DSLR camera (and give an advantage to the DSLR camera because Glass does not include a flash), a flash is rarely employed during autopsies or forensic examinations because it could easily cause overexposure at close distances and could also lead to reflections when photographing wet tissue during an autopsy.

Raters were asked to specify their opinion about each image using a Likert scale with 5 levels (ie, ++, +, +/-, -, and --, in which “++” represented a highly positive and “--” represented an extremely negative rating for the respective parameter. For statistical analysis, these values were transformed into numeric values, in which the highest possible rating “++” corresponded to 5 and the lowest rating “--” corresponded to 1.

In step 2, using the images acquired during the postmortem examinations, we were primarily interested in whether there were significant differences in how well users captured the desired anatomy when using either the DSLR camera or Glass. Because one device is handheld and the other is head mounted, the way users aim the device and shoot the image differs. Because there were small but perceivable differences in coloring (specifically, a very slight yellowish tint in the DSLR camera images), we decided to apply an automatic white balancing algorithm integrated into the GNU Image Manipulation Program (GIMP) 2.8 for all images of the postmortem examination. Based on the RGB color model, for each color channel, this algorithm discards pixel values at both ends of the histogram for the respective channel that contributes only to 0.05% of the image and stretches the remaining pixel values as much as possible. This procedure avoids undue influence of outliers at both ends of the spectrum for each of the channels [21]. For documentation purposes in forensics, this is a commonly applied method; thus, it does not add any steps. But, in our case, the algorithm was specifically applied because we did not want the raters’ decisions about which device was used for each of the presented images to be biased by the differences in tint. With respect to color, as stated previously, we were interested in whether the devices allowed adequate discrimination of colors. Independent of the device used for image acquisition, the algorithm was used on all images acquired during the postmortem examinations without informing the participants about this process before they gave their ratings. After performing the white balance for all 110 pictures of the postmortem examination, the images were loaded into the aforementioned app as described previously and presented to each of the participants in a randomized manner.

### Statistical Analysis

An observer recorded the time span (in seconds) required for each single postmortem examination using the stopwatch functionality available on a separate smartphone (Samsung Galaxy S4). The physicians had to start their examinations from a defined location on the bodies to be examined. A Wilcoxon signed rank test [22] was used to compare the related samples to detect differences between the devices. For each of the postmortem examinations, the number of images taken with the device chosen for that examination (Glass or DSLR camera) was counted. Before and after each use of Glass during an examination, the remaining battery charge was noted. For all mentioned variables, descriptive statistics were calculated, including the mean and standard deviation (SD).

The descriptive statistics for the ratings included the tabulation of the frequency and percentages of scale items for each item per device. Median values and quartiles were also calculated. To detect differences between the ratings obtained for both devices, we calculated an unpaired rank sum test (2-sided Mann-Whitney *U* test, with Cronbach alpha=.05 [23]). All items were included and there were no missing entries.

To determine interrater reliability (ie, how strongly the ratings of the participants correlated), we calculated intraclass correlation coefficients (ICC) [24] for the 6 participants for the items region of interest, sharpness, color, and brightness. The ICC can be used to assess the consistency of quantitative measurements (ie, correlation) between multiple observers measuring the same quantity [25]. All observers rated each case—they were not randomly chosen. We considered single values of the observers. We decided to use the ICC (3,1)-type, 2-way mixed with average measures for the calculations that were conducted using SPSS 21 (IBM Corp, Armonk, NY, USA).

### Institutional Review Board Approval

The study was conducted with approval by the Institutional Review Board of Hannover Medical School, study number 2069-2013.

## Results

### Image Acquisition

During the autopsy, 64 pictures were taken: 40 with Glass and 24 using the DSLR camera. A total of 15 images were excluded because they were not related to the deceased person’s anatomy, but rather to pictures of additional paperwork provided by the authorities. Thus, 49 autopsy images (Glass: n=32; DSLR camera: n=17) were used for the study. During the postmortem examinations, 112 pictures were taken, 55 of these using Glass and 57 using the DSLR camera. We excluded 2 pictures (for similar reasons); thus, 110 images (Glass: n=54; DSLR camera: n=56) remained for evaluation.

During postmortem examinations, an average number of 7 pictures were taken per case with both devices. When using the DSLR camera, the mean duration of a single postmortem examination was 225.9 seconds (SD 50.6) compared to Glass, for which the mean duration was 268.5 seconds (SD 64.1) (Table 1). During the postmortem examinations, an average 5.5% (SD 1.85) of Glass’ battery charge was used per case, corresponding to 0.81% per minute or 0.79% per picture.

**Table 1:** Descriptive statistics of time measurements, number of pictures taken (per case), and loss of battery charge (per case) during postmortem examinations, stratified by device used to capture image: digital single-lens reflex (DSLR) camera or Google Glass.

Usage statistics per device		Minimum	Maximum	Mean (SD)
**Time measurements (seconds)**				
	DSLR camera	189	310	225.9 (50.6)
	Glass	180	359	268.5 (64.1)
**Pictures taken**				
	DSLR camera	5	11	7.25 (2.12)
	Glass	4	9	7.0 (1.85)
**Used percentage of battery capacity per case (%)**				
	DSLR camera	N/A^b^	N/A	N/A
	Glass	3	8	5.5^a^(1.85)

^a^Battery decrease per minute: 5.5%/(268.5 s/60 s)=0.81%; per picture: 5.5%/7.0 pictures=0.79%.

^b^N/A: not applicable

### Interviews

Based on the interviews, we obtained subjective ratings of the user experience for both Glass and the Blink-app app. In the forensic setting, especially during the autopsy, Glass equipped with Blink-app was deemed as a suitable tool for examiners in situations where they needed both of their hands for fulfilling tasks, especially in cases where there is limited opportunity for other persons to take pictures (either because of space requirements or availability). This certainly holds true in autopsy settings. Another useful effect was that other persons attending the procedure (colleagues and a police officer) were directly able to review what the leading pathologist had seen by looking at the laptop’s screen (Figure 2). In the version that was tested, for users who do not have to wear corrective glasses, the device does not disturb the examiner’s sight during the procedure and is comfortable to wear because of its low weight and good ergonomics. Even in a busy environment, such as an autopsy with 5 people involved, the voice command used for starting the app worked very well. The integrated gesture control (nodding for upload and shaking one’s head for discarding an image) was perceived as natural.

**Figure 3:**
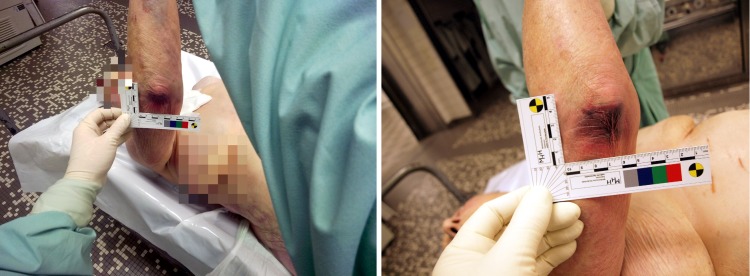
Similar phases of a postmortem on the same body with photo documentation (image anonymized for patient confidentiality). The image on the left was taken with Google Glass and the image on the right with the DSLR camera.

Similarly, for the postmortem examinations, both examiners agreed that the device’s ergonomics and light weight made it comfortable to wear. Because the environment is generally quieter when performing this kind of procedure, both the voice and gesture control worked well. The examiners mentioned that taking images using Glass took more physical effort than with the DSLR camera to capture the desired regions of interest (Figure 3). This was especially the case with close-ups because the device is placed on the head during use and there was no macro function available. Consequently, to obtain images of the region they wanted, they sometimes had to bring their head—and with it the device—closer to the findings than they would have preferred.

### Rating of the Images

The 6 raters evaluated 49 autopsy images each, totaling 294 evaluations (DSLR camera: n=102; Glass: n=192), each consisting of ratings on a 5-point Likert scale (from “++” to “--” corresponding to a numeric scale of 5 to 1) for each of the 4 qualities region of interest, sharpness, color, and brightness. Table 2 shows the absolute frequencies and percentages obtained per quality for each of the scale items for both devices. Differences between both devices are particularly noticeable for region of interest and sharpness. For region of interest, a large percentage of images taken by DSLR camera achieved the highest rating of “++” (region of interest: 57.8%, sharpness: 68.6%), whereas for images taken by Glass, ratings for these 2 qualities were more evenly distributed with a somewhat smaller peak for rating “+” (region of interest: 41.7%, sharpness: 34.9%). The distributions of ratings for color and brightness for both devices were a closer match, although results were also slightly in favor of images taken by DSLR camera.

For the images taken during the postmortem examinations (n=110), a total of 660 single evaluations were obtained from the 6 raters (DSLR camera: n=342; Glass: n=318). Each of the 4 qualities (region of interest, sharpness, color, and brightness) were rated using a 5-point Likert scale (from “++” to “--” corresponding to a numeric scale from 5 to 1). Table 2 shows the absolute frequencies and percentages obtained per quality for each of the scale items for both devices. Considering the percentage values for the scale items for sharpness, color, and brightness, ratings are only slightly in favor of the DSLR camera. For region of interest, the difference is considerable, as shown when looking at the sum of ratings for “+” and “++” (corresponding to positive and highly positive ratings), which amounted to 90.7% for images acquired using the DSLR camera vs 61% for those taken by Glass.

As mirrored by the median values and interquartile ranges (IQR) shown in Table 3, for both devices, the 6 raters were quite consistent in their evaluation of the 4 qualities (region of interest, sharpness, color, and brightness) for images taken during the autopsy (n=49). For the postmortem examinations, raters were again quite consistent in their evaluation of the 4 qualities (region of interest, sharpness, color, and brightness) (Table 3).

**Table 2:** Frequencies (n) and percentages (%) of evaluations given by 6 raters for images taken during autopsy (n=294) and postmortem examination (n=660) with a digital single-lens reflex (DSLR) camera and Google Glass.

Quality and rating scale^a^		Autopsy evaluations, n (%)		Postmortem examination evaluations, n (%)	
		DSLR camera (n=102)	Glass (n=192)	DSLR camera (n=342)	Glass (n=318)
**Region of interest**					
	++	59 (57.8)	56 (29.2)	161 (47.1)	82 (25.8)
	+	33 (32.4)	80 (41.7)	149 (43.6)	112 (35.2)
	+/-	7 (6.9)	35 (18.2)	28 (8.2)	92 (28.9)
	-	3 (2.9)	20 (10.4)	4 (1.2)	32 (10.1)
	--	0 (0)	1 (0.5)	0 (0)	0 (0)
	Total	102 (100)	192 (100)	342 (100)	318 (100)
**Sharpness**					
	++	70 (68.6)	45 (23.4)	160 (46.8)	146 (45.9)
	+	24 (23.5)	67 (34.9)	129 (37.7)	111 (34.9)
	+/-	6 (5.9)	37 (19.3)	37 (10.8)	46 (14.5)
	-	2 (2.0)	40 (20.8)	9 (2.6)	15 (4.7)
	--	0 (0)	3 (1.6)	7 (2.0)	0 (0)
	Total	102 (100)	192 (100)	342 (100)	318 (100)
**Color**					
	++	38 (37.3)	40 (20.8)	134 (39.2)	147 (46.2)
	+	52 (51.0)	86 (44.8)	152 (44.4)	88 (27.7)
	+/-	11 (10.8)	54 (28.1)	47 (13.7)	71 (22.3)
	-	1 (1.0)	12 (6.3)	9 (2.6)	12 (3.8)
	--	0 (0)	0 (0)	0 (0)	0 (0)
	Total	102 (100)	192 (100)	342 (100)	318 (100)
**Brightness**					
	++	29 (28.4)	32 (16.7)	124 (36.3)	129 (40.6)
	+	47 (46.1)	79 (41.1)	147 (43.0)	102 (32.1)
	+/-	21 (20.6)	61 (31.8)	61 (17.8)	52 (16.4)
	-	5 (4.9)	20 (10.4)	10 (2.9)	34 (10.7)
	--	0 (0)	0 (0)	0 (0)	1 (0.3)
	Total	102 (100)	192 (100)	342 (100)	318 (100)

^a^Rating “++” indicates a highly positive rating; “--”stands for very poor results.

**Table 3:** Median values and interquartile ranges (IQR) of evaluations given by 6 raters for images taken during autopsy (n=294) and postmortem examination (n=660) with a digital single-lens reflex (DSLR) camera and Google Glass.

Quality and participant		Autopsy evaluations^a^				Postmortem examination evaluations^a^			
		DSLR camera (n=102)		Glass (n=192)		DSLR camera (n=342)		Glass (n=318)	
		Median	IQR	Median	IQR	Median	IQR	Median	IQR
**Region of interest**									
	#01	5	1	4	1	5	1	5	1
	#02	5	1	4	1	5	1	4	1
	#03	5	0	5	0	5	0	5	1
	#04	4	0	4	1	4	0	3	1
	#05	4	2	3	2	4	0	3	1
	#06	4	1	3	2	4	2	3	1
**Sharpness**									
	#01	5	0	4	1.25	4	1	5	1
	#02	5	0	4	1	4	1	4	1
	#03	5	0	4	1	5	0	5	0
	#04	4	1	2.50	2	4	0	4	1
	#05	5	0	4	2	5	1	5	0
	#06	5	1	3	2	4	2	4	1
**Color**									
	#01	4	1	4	0	4	1	5	1
	#02	4	1	3	0.50	3	1	3	0
	#03	5	1	4	1	5	0	5	0
	#04	4	0	3	1	4	0	4	1
	#05	5	1	4	1	5	0	5	0
	#06	5	1	4	1	4	1	4	1
**Brightness**									
	#01	4	0	4	0	4	0	4	1
	#02	3	1	3	0	3	1	3	2
	#03	5	0	5	1	5	0	5	0
	#04	3	1	3	1.25	4	1	3	2
	#05	4	1	4	1	5	0	5	0
	#06	4	1	3	1	4	1	4	1

^a^For calculating median and IQR, the Likert scale items have been transformed into a numeric representation between 1 and 5 where 5 represents the best (“++”) and 1 the worst possible rating (“--”).

For image quality, it was of interest whether the region of interest was appropriately captured, whether the image was well focused and sharp, as well as whether color discrimination and brightness were satisfactory for the participants. Instead of using algorithms for an objective interpretation of the images, we decided to obtain subjective ratings by professionals who would have to use such images for their photo documentation if a device such as Glass were to be officially introduced in such settings. Overall, on a 5-point scale (where 5 represented the best and 1 the worst possible rating) the images received good (median 4) and very good values (median 5) in all 4 categories independent of the device used for image acquisition or the setting (Table 4).

**Table 4:** Minimum (min) and maximum (max) measurements, first quartile (Q1), second quartile (Q2, median), third quartile (Q3), and interquartile range (IQR) of ratings from 6 raters for images taken during autopsy (images: n=49, ratings: n=294) and postmortem examination (images: n=110, ratings: n=660) with a digital single-lens reflex (DSLR) camera and Google Glass.

Procedure and measurements		Region of interest		Sharpness		Color		Brightness	
		DSLR camera	Glass	DSLR camera	Glass	DSLR camera	Glass	DSLR camera	Glass
**Autopsy**									
	Min	2	1	2	1	2	2	2	2
	Max	5	5	5	5	5	5	5	5
	Q_1_	4	3	4	3	4	3	3	3
	Q_2_^a^	5	4	5	4	4	4	4	4
	Q_3_	5	5	5	4	5	4	5	4
	IQR^a^	1	2	1	1	1	1	2	1
**Postmortem examination**									
	Min	2	2	1	2	2	2	2	1
	Max	5	5	5	5	5	5	5	5
	Q_1_	4	3	4	4	4	3	4	3
	Q_2_^a^	4	4	4	4	4	4	4	4
	Q_3_	5	5	5	5	5	5	5	5
	IQR^a^	1	2	1	1	1	2	1	2

^a^For calculating median and IQR, the Likert scale items have been transformed into a numeric representation between 1 and 5 where 5 represents the best (“++”) and 1 the worst possible rating (“--”).

Pictures taken during the autopsy showed differences between DSLR camera and Glass primarily in region of interest and sharpness (DSLR camera: median 5; Glass: median 4). In color and brightness, the median values for both devices were identical (median 4), but values for the third quartile differed because only the DSLR camera received maximum ratings of 5. Nevertheless, images taken during the autopsy using Glass received significantly lower ratings for all 4 categories than those taken by the DSLR camera (region of interest: *z*=–5.154, *P*<.001; sharpness: *z*=–7.898, *P*<.001; color: *z*= –4.407, *P*<.001, brightness: *z*=–3.187, *P*=.001; see Table 5). Raters favored the pictures taken by DSLR camera with respect to correctly capturing the desired region of interest as well as sharpness, color discrimination, and brightness.

**Table 5:** Unpaired rank sum, 2-sided Mann-Whitney *U* (Cronbach alpha=.05) for ratings of autopsy and postmortem examination images taken by digital single-lens reflex (DSLR) camera and Google Glass.

Item	Autopsy (n=294)		Postmortem examinations (n=660)	
	*z*	*P*	*z*	*P*
Region of interest	–5.153691	<.001	–8390	<.001
Sharpness	–7.898378	<.001	–0.587	.56
Color	–4.406570	<.001	–0.011	.59
Brightness	–3.186663	.001	–540	.01

### Interrater Reliability

The interrater reliability was high (Table 6). The ICC for the ratings obtained based on the autopsy pictures indicated a strong positive relationship for sharpness (ICC=.917, 95% CI .875-.948, *P*<.001) and brightness (ICC=.720, 95% CI .579-.826, *P*<.001), and a moderately positive relationship between raters for color (ICC=.658, 95% CI .485-.787, *P*<.001) and region of interest (ICC=.630, 95% CI .443-.770, *P*<.001). When considering the ratings of the postmortem examinations, the ICC values for region of interest (ICC=.727, 95% CI .639-.799, *P*<.001) and sharpness (ICC=.761, 95% CI .685-.824, *P*<.001) indicate a strong positive relationship, whereas color (ICC=.674, 95% CI .569-.760, *P*<.001) and brightness (ICC=.545, 95% CI .399-.665, *P*<.001) indicate a moderately positive relationship among the raters.

**Table 6:** The intraclass correlation coefficients (ICC), 95% confidence intervals (95% CI), and levels of significance (*P*) for items rated by 6 raters for autopsy and postmortem examinations, ICC(3,1), 2-way mixed, average measure.

Item	Autopsy			Postmortem examinations		
	ICC	95% CI	*P*	ICC	95% CI	*P*
Region of interest	.630	.443-.770	<.001	.727	.639-.799	<.001
Sharpness	.917	.875-.948	<.001	.761	.685-.824	<.001
Color	.658	.485-.787	<.001	.674	.569-.760	<.001
Brightness	.720	.579-.826	<.001	.545	.399-.665	<.001

##  Discussion

### Principal Findings

**Figure 4:**
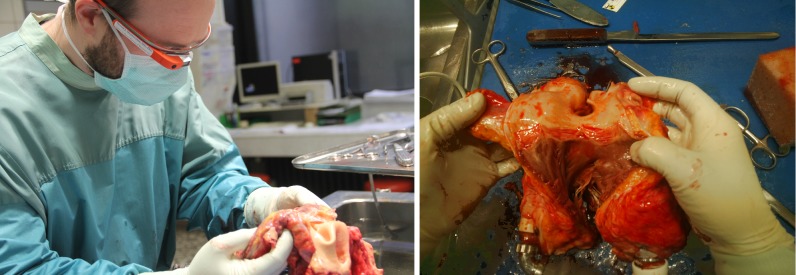
The forensic pathologist is taking a picture of the heart using Google Glass (left). The original image taken with the device allows viewers a first-person perspective of the structure (right).

In our evaluation, we focused on the main functionality of Glass in areas where we thought it would have an advantage against other existing technology. Because it includes a lightweight head-mounted display and a camera with voice and gesture control in combination with embedded computer and wireless communication technology, it seems ideal for both communication and photo documentation (Figure 4). We were interested in its effectiveness, efficiency, and user satisfaction.

Overall, both devices had sufficient capabilities and could be used effectively for the task of image acquisition during the procedures. Users were able to successfully achieve their objectives with either device, but we detected differences in the efficiency of the devices. Postmortem examinations performed using Glass for acquiring the photo documentation took 42.6 seconds longer than those performed with the DSLR camera (Table 1). The difference was significant. (*z*= –2.100, *P*=.04 using Wilcoxon signed rank test). Overall, this corresponds to one-fifth of the average length of the procedure when using a DSLR camera (3.45 min) and leads to additional undesired expenditure of battery power. On average, 5.5% (SD 1.85) of the battery capacity of Glass was used per case during the postmortem examinations, corresponding to 0.81% per minute or 0.79% per picture.

Although one may suspect that this relatively high power consumption may be because of not using the stock camera app for capturing the images, we do not believe this to be the case. While developing the app, we did not note any differences in power consumption when comparing our app to the preinstalled camera app. As noted previously, the Blink-app app was implemented based on the official camera API calls; thus, both apps made use of the same (or at least very similar) calls to image acquisition functions. Also, when using the Blink-app app, images were saved in the original form available through the official API calls without any alteration. Of course, power consumption is higher with an active Wi-Fi connection, but again, we noted no apparent difference between the stock camera app and the Blink-app app if the device was connected to a wireless hotspot while either app was running. Also, because we were dealing with a premarket device, we expect power consumption and battery capacity to improve in future versions. At the time of our study, our aim was simply to determine whether, in its current state, power consumption was a potential limiting factor or was adequate for our chosen setting without having to resort to external power sources, such as additional battery packs, because these would be problematic in such settings due to external cabling and additional bulk. In medical settings, a cable running down from Glass to an external battery pack may raise concerns with respect to hygiene as well as add potential for Glass to be pulled off the user’s nose if something (eg, a fastener on the physician’s surgical gown) inadvertently pulled on this cable.

For the DSLR camera, it was not possible to determine the exact percentage of battery power used because the camera only provided an icon with a crude scale in units of 25%; however, no significant loss of battery power could be detected. This is understandable when considering the average battery capacity of current DSLR cameras; thus, no corresponding information is listed in Table 1. For Glass, the battery’s level of charge quickly decreased; an average 5.5% of the battery’s capacity was spent per postmortem examination (0.8% per minute or 1.3% per picture). If all went well and examiners were to use Glass in a similar manner to how it was used in our study, this would allow for approximately 18 postmortem examinations or 125 minutes of use, or 78 pictures. Compared to the DSLR camera, this would require earlier recharging, which could be a problem if there were a large number of postmortem examinations to be performed. But it is expected that a future version of Glass sold to the general public will have an improved battery life. Altogether, the effort necessary for achieving the objectives was higher when using Glass compared to the DSLR camera, and when comparing the percentage of battery charge used, the numbers currently seem to be also in favor of the DSLR camera.

The presentation of the region of interest is the most important aspect that must be covered by the photographer and the device used. For the images taken during the forensic examinations, we decided to apply an automatic white balancing algorithm as described previously to give the images taken by both devices a similar and comparable appearance and to allow the participants to more easily focus on possible differences in the presentation of the region of interest. As expected, the median of the ratings for images taken with Glass or DSLR camera showed lower variability in the median (median 4) in all item categories because of the image manipulation. The IQR showed greater spans in the Glass group (IQR 2) with respect to region of interest, color, and brightness. Based on statistical testing, only the ratings for region of interest (*z*=–8.3901, *P*<.001) and brightness (*z*=–540, *P*=.01) were significantly better for images taken with the DSLR camera, whereas sharpness and color showed no significant differences (Table 5). The main disadvantage concerning region of interest is the lack of a zoom function with Glass compared to the DSLR camera.

User satisfaction depends on a number of factors, including the usability of the device and the quality of the images. The interviews about user experience and acceptance of the Glass device underline the comfort of a lightweight voice- and gesture-controlled device with a head-mounted camera. Those questioned perceived the device to be a suitable tool in the situations where they had used it (ie, during autopsy and forensic examinations).

Overall, the experience using Glass was satisfactory although the quality of the images obtained left room for improvement. Our use of the custom app instead of the device’s stock camera did not appear to have an influence on image quality. While developing the app, a careful visual comparison of images taken with both apps (taken by the same person with same angle of view and similar lighting conditions because the images were taken at the same location and only a few seconds apart) did not show any obvious differences in color, sharpness, and other parameters. There were also no visual differences with respect to artifacts or depiction of fine structures. Additionally, an analysis of the exchangeable image file format (Exif) data included within the images did not show any notable differences (aside from the timestamp). Therefore, we believe that the quality of images acquired by Glass was not negatively influenced by our app. However, it is to be expected that the manufacturer will refine many of the points we noted (ie, image quality and battery capacity) before the device hits the consumer market.

### Limitations

There are a number of limitations in our evaluation. First of all, because of time constraints, we were only able to use the device on a limited number of cases and with only 2 physicians. Also, for our study, the 6 raters included the 2 physicians who took the images, but because of the amount of images taken from similar perspectives with both devices by both physicians and some days having passed between image acquisition and evaluation, we do not believe this influenced the results. When asked, neither of them was able to identify who had taken a specific image because of the random manner in which the images were presented. In most cases, they were not even sure which case an image belonged to. Also, neither of the physicians had any influence on the integration of the selected images in the app we used for image presentation.

Because it was only possible to use Glass during one autopsy, we may have missed differences one might otherwise note with respect to specific types of cases that require other approaches than those commonly used in standard autopsy situations. Establishing a control group could also have improved the results. A larger number of postmortem examinations, ideally performed by additional examiners, would have reduced bias and standardized instruments for measuring usability could have been applied as well (eg, the system usability scale by Brooke [26] and Hassenzahl’s AttrakDiff2 [27]). Raters recruited from other forensic medical facilities would also have improved the data pool used for analysis.

From a technical point of view, it may be seen as problematic to compare the capabilities of a DSLR camera with a resolution of 12.6 megapixels with those of a 5-megapixel camera integrated in a mobile device. Even with recent advances in mobile technology, the quality of a small-lensed camera can never compare to what a DSLR camera has to offer. However, our aim was not a direct comparison of technical parameters but to determine whether the perceived image quality provided by Glass was adequate for the stated purpose; therefore, we do not believe this to be a limitation.

### Additional Advice

There are additional aspects that will make the deployment of Glass in clinical settings complicated. These expected complications are not due to purely technical issues, such as image quality or handling, but rather concerns about data protection and privacy. Because Glass was developed primarily for the private sector, its basic functionality and the preinstalled apps make extensive use of Google’s network and servers. Therefore, the user has little control over the way data are handled, transmitted, stored, and possibly evaluated by a third party. Because Google is the main company involved, their data protection policy is applied. For the professional medical sector, it is not advisable to send any data of a patient—especially concerning medical issues—using an open and unsecured network. At least in European countries, it is not acceptable to store and share medical data stored in “the cloud” and similar restrictions apply to other countries (eg, the US Health Insurance Portability and Accountability Act does not allow a third party to access patient data [10]). Therefore, unencrypted communication or communication over insecure networks for making video calls or doing live chats to exchange information about a patient’s case are just as unacceptable as sending pictures and emails containing personal details that help identify the patient.

Currently, when using Glass in a medical setting, a private (closed) network without any connection to servers aside from those belonging to the private infrastructure should be ensured. Additionally, just as for all other applications where medical data are concerned, state-of-the-art encryption and access policies should be employed for access to the infrastructure as well as storage and transmission of all data. For our study, we developed the previously mentioned app that allowed data exchange only within a private network (ie, between Glass and a laptop without Internet access).

There is also an additional point that must be kept in mind regarding apps running on Glass. Just as for other mobile smart devices used in health care settings, there will be additional pitfalls regarding data security and privacy once the market of third-party health apps and medical apps specifically adapted to Glass grows; for example, if manufacturers of apps do not implement appropriate measures for ensuring these aspects in their products. Depending on the jurisdiction they are used in, applications that have a diagnostic or therapeutic purpose (ie, could be rated as a medical device) must already conform to regulations (eg, the Mobile Medical Applications Guidance for Industry and Food and Drug Administration Staff [28]), thus ensuring some quality control. Nevertheless, for apps for which such regulations do not apply, it would be highly desirable if manufacturers or developers of an app were to provide users with at least sufficient and transparent information to allow them to make an informed decision about whether they want to use an app—be it on Glass or on other mobile devices—or not. The information should be provided in a clearly structured way (eg, using an app synopsis as presented by Albrecht [29]). This could also be used as a starting point for instigating a peer-review process of such applications.

Devices such as Glass can be used unobtrusively without attracting attention of persons who are unaware of the technical possibilities or dangers offered by these devices. As in all areas of medicine, the usual rules regarding a patient’s rights of privacy need to be applied, which may easily be overlooked because Glass is so easy to use and integrates well in many settings. Also, because it is currently unknown how living patients will react to the device in an examination and other common settings encountered in the medical field, empirical studies that investigate the acceptance of patients would add significant value if Glass is to be widely deployed. For example, a recent blog entry by Mat Honan [30] who used the device for a year indicated issues with acceptance from the public.

Hygiene is also an issue when using the device with patients because Glass is not a medical product and was not developed for use in a sterile environment. Therefore, it is questionable whether it is possible to disinfect it properly. Currently, the manufacturer is not giving any recommendations regarding proper disinfection of the device. By allowing hands-free operation, our Blink-app app supports the demands of hygiene; nevertheless, we used disinfecting wipes for plastic surfaces of medical devices, although this may cause issues with warranty [31].

### Conclusions

In our opinion, aside from using the device for documentation purposes, there is also potential for another field of application in forensic medicine: By chance, we recognized the reaction of our medical students participating in the autopsy. They were amazed by the pictures taken by Glass that were almost instantly presented on the laptop’s screen. Because there is only room for a limited number of students to watch, even for interesting cases, using the described setup with our additional add-ons might provide an opportunity for a larger number of students to observe the procedure in a dynamic manner. There are also a number of other uses in medical education and training one can imagine (eg, cardiology and others described in [32]).

The main strength of Glass is its ergonomic and lightweight design, combined with the camera that allows taking pictures and videos directly from the user’s point of view. This feature makes the design interesting for medical education (eg, in pathology and in surgery). Independent of the local setting, students and colleagues may join a complicated autopsy or operation and can see exactly what their teachers see, thus learning from watching their actions from the ideal perspective (Figure 4). This is similar to the potential benefit also already noted—albeit for education in general—by other authors [32-34]. If worn by students, Glass could also serve to enhance existing augmented reality-based solutions for medical education, such as mARble [35], an augmented reality-based blended learning tool for use in medical education that is currently implemented for conventional smartphones and allows students to immerse themselves in an almost realistic learning scenario in cases where learning on real patients may be restricted (eg, because of ethical concerns). It remains to be seen whether the current comparatively low display resolution of Glass is sufficient to provide students with content similar to what can currently be shown using the smartphone-based solution (eg, by overlaying specific medical findings on another student’s skin). If yes, Glass would also have potential to significantly enhance augmented reality-based learning tools.

## Abbreviations

API: application programming interface
DSLR: digital single-lens reflex
Exif: exchangeable image file format
ICC: intraclass correlation
IQR: interquartile range
Q1: first quartile
Q2: second quartile (median)
Q3: third quartile
